# Evaluating Oregon's occupational public health surveillance system based on the CDC updated guidelines

**DOI:** 10.1002/ajim.23139

**Published:** 2020-06-01

**Authors:** Liu Yang, Crystal Weston, Curtis Cude, Laurel Kincl

**Affiliations:** ^1^ School of Biological and Population Health Sciences, College of Public Health and Human Sciences Oregon State University Corvallis Oregon; ^2^ Public Health Division Oregon Health Authority Portland Oregon

**Keywords:** occupational health indicators, occupational safety and health surveillance, surveillance evaluation

## Abstract

**Background:**

The Oregon Occupational Public Health Program (OOPHP) monitors occupational health indicators (OHIs) to inform occupational safety and health (OSH) surveillance. In 2018, OOPHP evaluated the performance of the OSH surveillance system and identified areas for future improvement.

**Methods:**

Following the Centers for Disease Control and Prevention (CDC) updated guidelines for evaluating public health surveillance systems, the OOPHP evaluation team engaged internal and external stakeholders using a mixed‐methods approach. Operational measures for ten surveillance attributes were developed. Multiple data collection methods resulted in credible evidence for evaluation conclusions. Analyses included summary statistics and qualitative analysis of interviews, a focus group, and online surveys.

**Results:**

Twenty stakeholders took part in this evaluation, with an average participation rate of 55%. Results showed the Oregon OSH surveillance system was simple, flexible, and highly accepted by its stakeholders. Funding security presents challenges for stability. A lack of timeliness of OHIs, low relevance of OHIs to local OSH issues, and the system's ineffectual data dissemination all limit the usefulness of the OSH surveillance system. A review of key data sources for the system showed good data quality and predictive value positive, but relatively poor sensitivity and representativeness.

**Conclusions:**

The evaluation team successfully adapted attributes and examples in the CDC guidelines to this Oregon OSH surveillance evaluation. The evaluation findings have informed the development of recommendations for improvements to OOPHP's OSH surveillance. Future research is needed to develop guidance specific to OSH surveillance evaluation.

## 1 INTRODUCTION

Public health surveillance is the ongoing, systematic collection, analysis, interpretation, and dissemination of data regarding health‐related events for use in public health action.^1^ Occupational safety and health (OSH) surveillance is an important type of public health surveillance that collects data on work‐related fatality, injury, and illness and the presence of workplace hazards. OSH surveillance activities were formalized in the United States in the 1970s with the enactment of the Occupational Safety and Health Act.^2^ The National Institute for Occupational Safety and Health (NIOSH) under the Centers for Disease Control and Prevention (CDC) supports national and state‐level OSH surveillance programs.^3^ Currently, NIOSH funds 26 states to conduct state‐level OSH surveillance programs.

In the long‐term, NIOSH envisions that all states will have the capacity to conduct OSH surveillance and contribute to national, state, and local prevention efforts.^3^, ^4^ To strengthen states’ OSH surveillance capacity, the Council of State and Territorial Epidemiologists (CSTE) occupational health surveillance workgroup in collaboration with NIOSH developed and has been updating occupational health indicators (OHIs) as the minimum state surveillance capacity since early 2000s.^4^, ^5^, ^6^, ^7^ OHIs is a set of measures of prioritized OSH conditions covering work‐related injuries and illnesses, exposures, hazards, intervention efforts, and socioeconomic impacts. As of 2018, 24 OHIs have been developed for use by states.

The Oregon Occupational Public Health Program (OOPHP), established in 1987, is currently funded by NIOSH to conduct expanded state‐level OSH surveillance. The objective of OOPHP is to reduce work‐related injury, illness, and death through surveillance, investigation, analysis, and development and distribution of prevention recommendations in Oregon. OOPHP's OSH surveillance system tracks all the 24 OHIs using 19 different data sources.

In 2018, OOPHP collaborated with Oregon State University (OSU) to conduct a comprehensive evaluation of its OSH surveillance system. The evaluation followed the *Updated Guidelines for Evaluating Public Health Surveillance Systems* published by the CDC^1^ (hereinafter called the *CDC Updated Guidelines*) to understand the system's performance and to identify gaps for future improvement. The *CDC Updated Guidelines* are by far the most well‐known and the de facto authoritative guideline for public health surveillance evaluation. It is intended to be universally applicable to the great variety of public health surveillance systems.

This paper describes the evaluation process, results, and lessons learned and offers recommendations for improvement of OOPHP and evaluation methodologies for OSH surveillance systems. This evaluation is of particular interest because there have been few published evaluations on state‐level OSH surveillance systems in the US. Gaps and experience learned from evaluating the Oregon OSH surveillance system can help to improve other state‐level OSH surveillance systems and programs as well as their evaluation.

## 2 METHODS

*CDC Updated Guidelines* provide generic recommendations for evaluation of public health surveillance systems but lack detailed information needed to guide the evaluation process.^8^, ^9^, ^10^ Particularly, it lacks specifics pertaining to the surveillance of occupational health. As a result, the evaluation team had to develop a detailed methodology for evaluating the Oregon OSH surveillance system based on the general principles in the guidelines, including methods for engaging stakeholders and collecting data.

The overall evaluation approach followed the six tasks recommended in the *CDC Updated Guidelines*:

*Describe the surveillance system and determine the scope of work*: Information on the system's work process, surveillance methodology, data sources, organizational structure, and IT infrastructure was collected through a thorough review of the system's working documents, onsite observation, and communication with program leadership and staff. An evaluation team, comprised of evaluators from OSU and the program's leadership and staff, determined the scope of work through formal discussions.

Given limited time and resources for the evaluation, the evaluation team selected three key OHI data sources over which OOPHP might have influence, the inpatient hospital discharge (HD) data, the disabling workers’ compensation (WC) data, and the adult blood lead epidemiology and surveillance (ABLES) data were chosen for assessment. A list of these three key data sources and the corresponding OHIs that are calculated from the data sources is shown in Table 1.

**Table 1:** Key data sources and corresponding occupational health indicators (OHIs)

Data source	Corresponding OHIs
Hospital Discharge (HD) data	Work‐related hospitalizations
	Hospitalization for work‐related burns
	Hospitalization from or with pneumoconiosis
	Work‐related low‐back disorder hospitalizations
Workers' Compensation (WC) data	WC claims for amputation with lost work‐time
	WC claims for carpal tunnel syndrome with lost work‐time
Adult Blood Lead Epidemiology and Surveillance (ABLES) data	Elevated blood lead levels among adults

*Identify and engage stakeholders*: Based on a thorough understanding of the Oregon OSH surveillance process, the evaluation team identified major internal and external stakeholders from OSH regulatory, academic, public health, and WC organizations. The team grouped representatives into program leadership including higher‐level leaders and the program's management and key personnel, key surveillance staff, external experts, data providers, disseminators, and users. Stakeholders were further ranked into three levels based on their involvement with the system to facilitate the design of the evaluation approach. To inform and engage stakeholders, the evaluation team gave formal presentations and reached out by email to introduce the evaluation project and describe the data collection methodology.

*Develop the evaluation approach*: *CDC Updated Guidelines* recommend 10 surveillance attributes for assessing a surveillance system's data quality and performance. The evaluation team sorted them into three categories: performance (simplicity, flexibility, acceptability, timeliness, and stability), data quality (data quality, sensitivity, predictive value positive [PVP], representativeness), and overall usefulness. For performance and overall usefulness attributes, the evaluation focused on the whole OOPHP and its OSH surveillance system, while for data quality attributes, the evaluation was limited to the three key data sources and associated OHIs (Table 1). A core task in the evaluation was to design a practical evaluation approach for assessing the ten attributes. The evaluation team referred to both the *CDC Updated Guidelines* and other surveillance evaluation literature to develop a set of operational measures for assessing each attribute and to specify data collection and analysis methods for each measure (Table 2).^1^, ^11^, ^12^, ^13^, ^14^ Five main data collection methods were used in this evaluation, including semi‐structured interviews, a focus group discussion, online surveys, a comprehensive document/literature review, and onsite observations. The best methods were selected for each measure to collect appropriate information. For example, we conducted focus group and interviews among the system's leaders and key personnel to solicit in‐depth discussions on the system's flexibility, stability, and usefulness, while sought only general perspectives in an online survey on a few attributes such as acceptability and usefulness from external experts and other stakeholders with a low level of involvement in the program. Table 3 shows the data collection method, type of participants, and the corresponding attributes for which evaluation evidence was collected.

**Table 2:** Approaches for evaluating surveillance attributes

Attribute	Definition	Evaluation measure	Evidence collection method^a^	Evidence collected/data analysis
System performance				
Simplicity	Structure and ease of operation to perform its stated objectives	(1) Data sources needed	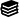	Types & number of data sources needed
		(2) Ease of obtaining and processing data	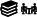	Work process
		(3) Ease of event/case ascertainment	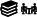	Rating of ease; possible challenges/problems
		(4) Number of organizations requiring data reports	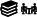	Types of data reporting & number of organizations
Flexibility	Ability to adapt to operating conditions or informational changes with little additional time, personnel, or funds	(1) Whether the system accommodates updates and changes in OHI methodology?	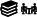	Past examples; potential changes and the system's preparation
		(2) Whether it accommodates other changes (eg, funding, data sources, technologies and standards, policies and regulation, emerging OSH issues)?	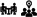	Past examples; potential changes and challenges; the system's preparation; resources available
Acceptability	The willingness of persons and organizations to participate in the surveillance system	(1) Willingness of stakeholders to collaborate with the program	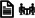	Rating of willingness; possible barriers & problems
		(2) Stakeholders awareness of the OSH surveillance system's objectives	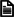	Summarize statistics
		(3) Stakeholders' participation in program activities	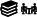	Participation rate; collaboration process & collaborators’ responsiveness
Timeliness	The speed between steps in a public health surveillance system	(1) The time gap between the occurrence of a case/event and the report of OHI	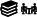	Time gap (in month/year); possible reasons & barriers
		(2) The amount of time spent for each working step in the system	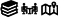	Time spent for each step in the logic model; possible reasons & barriers
		(3) Stakeholders' perspectives on the time lag	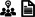	Stakeholders' perspectives; discuss OHIs/events that should/could be tracked more timely
Stability	System's reliability (without failure) and availability	(1) Little or no failure in operating the system	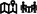	Working environment and infrastructure; past serious failures and possible current issues
		(2) Being operational when needed		
	System's sustainability	(3) Financial resources (funding support)	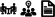	Possible issues with funding & other resources; availability for continuous resources and support
		(4) Other resources (human, technical, leadership support) to sustain the system		
Data quality (for key data sources)				
Data quality	Completeness and validity of the data	(1) Data validity (is the case/event correctly measured what it is intended to measure?)	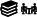	Possible validity issues
		(2) The completeness of the source data (less % of "unknown" or "blank" responses)	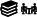	Completeness rate if possible; possible issues
		(3) Data quality control process in place to monitor errors/avoid missing data	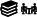	Data quality control process & problems
Sensitivity	The proportion of cases/events detected by the surveillance system	(1) The proportion of cases/events detected by each OHI	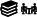	Report quantitative data if possible; potential issues
	The ability to monitor changes in the number of cases over time	(2) Ability to monitor changes over time	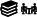	Past examples; key staff's perspective
		(3) Any active surveillance approaches used	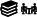	Active surveillance approaches & their effects on sensitivity
Predictive value positive (PVP)	The proportion of true cases among all reported cases	(1) The proportion of true cases among all reported cases	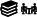	Report quantitative data if possible; potential issues
		(2) Approaches for confirming true cases	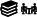	Approaches & their effects on PVP
Representativeness	The ability of the surveillance system to accurately describe the occurrence and distribution of a health‐related event by time, place, and person	(1) Is denominator used appropriately for OHIs to match numerator?	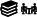	Possible issues with the choice of denominators
		(2) Is there any subpopulation excluded from the OHI?	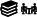	Subpopulations potentially excluded; Statistics whenever possible
Overall usefulness				
Usefulness	Contribute to the prevention and control of adverse occupational health conditions	(1) Contribute to the prevention and control of adverse occupational health events and to an improved understanding of the public health implications of such events	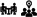	Ways to use OHI data; ways to promote dissemination & usage
		(2) Relevance & significance of the system's objectives and activities to the OSH needs to be perceived by stakeholders	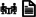	Stakeholders’ ratings & comments
		(3) Overall usefulness of the surveillance system perceived by stakeholders	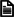	Stakeholders’ ratings & comments

Abbreviations: OHI, occupational health indicator; OSH, occupational safety and health.

^a^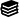 Document/literature review; 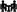 interview; 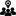 focus group; 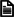 survey questionnaire; 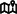 onsite observation.

**Table 3:** Data collection methods and corresponding stakeholders and attributes

Evidence collection method	Stakeholder group	Level of involvement	Format	Targeted attributes	Participated	Participation rate (%)
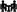 Interview	Higher level supporting leaders	Level 3	Phone call	Stability, Usefulness	3	100
	Key OSH surveillance staff	Level 1	In‐person	Simplicity, Flexibility, Acceptability, Timeliness, Stability, Data quality, Sensitivity, PVP, Representativeness, Usefulness	1	100
	Key data source provider	Level 2	In‐person/phone call	Flexibility, Timeliness, Stability, Data quality, Sensitivity, PVP, Representativeness	4	100
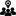 Focus group	Management and key personnel	Level 1	In‐person	Flexibility, Timeliness, Stability, Usefulness	7	87.5
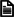 Survey	Management and key personnel	Level 1	Online	Flexibility, Acceptability, Timeliness, Stability, Usefulness	6	75
	External experts	Level 2	Online	Flexibility, Acceptability, Timeliness, Stability, Usefulness	4	25
	All other stakeholders	Level 3	Online	Acceptability, usefulness	4	36.4
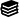 Document/literature review	…	…	Working documents/published literature	Simplicity, Flexibility, Acceptability, Timeliness, Data quality, Sensitivity, PVP, Representativeness	/	/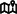 Onsite observation	…	…	Onsite	Timeliness, Stability	/	/

Abbreviations: OSH, occupational safety and health; PVP, predictive value positive.

*Gather credible evaluation evidence*: Based on the above specified methods, the evaluation team developed data collection protocols including interview and focus group guides, and survey questionnaires (Supporting Information Appendix). All data collection guides and questionnaires were pretested by more than three evaluators and researchers in OSU.

Semi‐structured interviews were conducted by a phone call or in‐person depending on the participants’ convenience. The focus group discussion was conducted in‐person. The online surveys were delivered via Qualtrics. Stakeholders’ participation and data collection were carried out from May to July 2018. The lead author (LY) conducted a review of working documents and published literature and onsite observations of routine operations throughout the evaluation process.

*Analyze collected evidence and make conclusions*: Interviews and focus group discussions were audio‐recorded, transcribed, and coded for themes. Mixed methods were used for data analysis. Qualitative summaries were reported by reviewing evaluation evidence collected from different sources, with quantitative statistics used whenever possible.

For system performance and the overall usefulness, judgments were reached by consensus of the evaluation team for each attribute. To assess overall data quality, the evaluation team rated each measure of the data quality attributes on a 5‐point scale, with 1 indicating the worst quality and 5 the best quality. Average ratings were calculated for each attribute and each key data source. An overall average score was then calculated to quantify the system's data quality.

*Ensure the use of evaluation findings*: Evaluation findings were reported to the OOPHP leadership and its advisory committee through a few meetings. Possible recommendations and feasible action plans were discussed to promote feasible recommendations.

No ethics review and approval were required because the project was regarded as evaluation instead of research.

## 3 RESULTS

Twenty stakeholders took part in 28 data collection sessions with an average participation rate of 55% (see Table 3 for the number of participants in different sessions). The participation rates in interviews, a focus group and online surveys were 100%, 87.5%, and 38.9%, respectively. For stakeholders from level 1 to level 3 (with 1 representing the highest level of involvement in the system and 3 the lowest), the participation rates were 82.4%, 100%, and 29%, respectively. More than 100 different working documents including work flowchart and logic model, organizational chart, program grant and surveillance protocols, working records and surveillance reports, as well as published literature were reviewed. Multiple onsite visits were performed as needed.

### 3.1 The system's performance

A detailed assessment of the five attributes to determine the Oregon OSH surveillance system's performance is shown in Table 4.

**Table 4:** Evaluation results for performance attributes

Attributes	Evaluation measure	Evidence collected	Assessment	Overall evaluation
Simplicity	(1) Data sources needed	>15 secondary data sources needed No data reporting & recording components	Very simple	Very simple
	(2) Ease of obtaining and processing data	Obtain only aggregated data for 60% OHIs Five data sources (including the three key data sources) involve straightforward data processing	Easy and straightforward	
	(3) Ease of event/case ascertainment	Average rating of ease by key staff: 3.7 of 5‐point scale (5 represents the easiest), with 13 OHIs rated 4 or 5 Six out of the 24 OHIs under surveillance were rated below 3, with difficulties related to data interpretation, meticulousness in data processing, etc	Easy	
	(4) Number of organizations requiring data reports	Only the grant office requiring reports	Very simple	
Flexibility	(1) Accommodates updates and changes in OHI methodology	Adapts changes in OHI annual guide very well, such as adding new OHIs and using alternative data sources	Very flexible	Flexible
	(2) Whether it accommodates other changes	Leadership is an awareness of new trends and challenges Past examples attempted to respond to local OSH surveillance needs	Has the potential to adapt changes	
Acceptability	(1) Willingness of stakeholders	Average willingness rating was 4.8 86% participants chose “very willing” (rating 5)	Very willing	Highly accepted
	(2) Stakeholders’ awareness of the system's objectives	93% participants were aware of the system's objectives	Stakeholders held positive perspectives towards the system	
	(3) Stakeholders' participation	Average attendance rate in advisory committee board meetings was 60% Stakeholders were responsive in work collaboration	Good	
Timeliness	(1) The time gap of OHIs	2‐3 years lag	Not timely	Not timely
	(2) The amount of time for working steps	Data collection and report process was timely Dissemination speed cannot be assessed as little dissemination work done	Timely	
	(3) Stakeholders' perspectives	70% participants accepted the time lag; OHIs limited in their usability being lagging indicators;	Fair, but limited in usability	
Stability	(1) Reliability	Able to produce OHIs efficiently and effectively	Very stable	Stable
	(2) Availability	Able to generate products when needed	High availability	
	(3) Financial resources	Funding a big concern for sustainability; NIOSH seemed to be the only funding source The competitive research application proposal is challenging	Lack of long‐term funding security	
	(4) Other resources	Received support from its housing agencies and partners All stakeholders are very willing to collaborate with the program	Good	

Abbreviations: NIOSH, National Institute for Occupational Safety and Health; OHI, occupational health indicator; OSH, occupational safety and health.

*Simplicity*: The Oregon OSH surveillance system is simple, without complicated surveillance design for data collection, processing, and case definition. The work process is straightforward.

*Flexibility*: The OHI methodology guide is regularly updated to add new OHIs or adjust data sources of existing OHIs to reflect changes in the field. The system displays high flexibility in adopting these changes since 2004 when it started to track OHIs.^15^ We identified past examples that showed the system's flexibility to respond to local OSH needs. For instance, a “Story Map” project in 2018 produced OHI for local use based on county‐level data and state list of hazardous industries.^16^

*Acceptability*: The system was rated as highly accepted. The average willingness of stakeholders to collaborate with the system was rated as 4.8 on a 5‐point scale, with 1 indicating the least accepted and 5 the most accepted. Stakeholders were actively involved in the system's activities.

*Timeliness*: Although the Oregon OSH system can produce OHIs in a timely fashion once data are available, there was a 2‐ to 3‐year gap between the occurrence of an occupational health event or case and the generation of a corresponding OHI. For example, the 2015 OHI report was produced in mid‐2018.

*Stability*: System stability was measured with three indicators: reliability, availability, and sustainability. High reliability and availability of the Oregon OSH system are demonstrated by the successful production of OHIs and related working reports. However, long‐term funding security did present challenges to the system's sustainability. Competitive NIOSH grant funding is the only funding source for the OOPHP. Opportunities for alternative sources were not readily identified. As the system's leadership and key staff responded, if the OOPHP could not successfully renew its NIOSH funding, “there would be no such program in Oregon.”

### 3.2 Data quality

Four attributes (data quality, sensitivity, PVP, and representativeness) were used to assess data quality. Table 5 summarized results for each measure and for each of the three key data sources (the inpatient HD data, disabling WC data, and ABLES data).

**Table 5:** Evaluation results for data quality attributes

Attribute	Evaluation measure	HD data		WC data		ABLES data		Average score
		Evidence collected	Score	Evidence collected	Score	Evidence collected	Score	
Data quality	(1) Data validity	Key definitions were scientifically sound	5	Key definitions were scientifically sound; Some variables (eg, injury date) were not precise for chronic injuries and illnesses.	4	BLL was confirmed by lab test	5	4.7
	(2) Completeness	Missing rate in “primary payer” was considered to be low Literature concerned on miscoding & omission of comorbidities, which may affect three of the four associated OHIs^7^, ^17^, ^18^	4	Missing rates were 0% for injury nature, up to 4% for injury event in 2016‐2017;	5	Missing rate of some variables (eg, age) was considered to be low^19^ Complete and correct residency information: 71% (among cases needing mail follow‐up, 2016‐2018 data)	4	4.3
	(3) Data quality control process	Standard data quality controls^20^ Literature showed concerns with data quality control^17^	3	Formal internal quality control process for coding and check errors	5	Standard data quality controls Follow‐up investigation helped to confirm/correct missing/wrong residency and other information	4	4.0
Average score (data quality)		/	4.0	/	4.7	/	4.3	4.3
Sensitivity	(1) True cases/events detected	Certain hospitals were excluded WC under‐coverage & under‐reporting issue (see WC data section) Errors & misclassifications existed^7^, ^17^, ^18^	3	Certain populations were excluded^21^ Literature reported under‐reporting as a common problem, especially for illnesses^22^, ^23^, ^24^	3	Mandatory BLL testing facilitated case identification, but certain populations were excluded^25^	4	3.3
	(2) Monitor changes	Coding changes caused “break‐in‐series”^17^	4	Coding changes caused “break‐in‐series”^7^	4	Definition changes caused misclassifications^26^	3	3.7
	(3) Active surveillance approaches	No active approach	3	No active approach	3	Follow‐up investigation could help to identify potential cases	5	3.7
Average score (sensitivity)		…	3.3	…	3.3	…	4.0	3.6
Predictive value positive (PVP)	(1) Proportion of true cases	Misclassifications was a concern^7^, ^17^, ^18^	4	Insurers’ review was regarded as effective Misclassifications in data coding & entry were minimum	5	Definition changes caused misclassifications (ie, false‐positive incidences)^26^	4	4.3
	(2) Approaches for confirming true cases	No approach in place	3	The insurers investigated cases for correct information	5	Follow‐up investigation could help to correct errors	5	4.3
Average score (PVP)		…	3.5	…	5.0	…	4.5	4.3
Representativeness	(1) Is denominator used appropriately?	Denominator/baseline populations match the numerators	5	Denominator/baseline populations match the numerators	5	Denominator/baseline populations match the numerators	5	5.0
	(2) Any subpopulation excluded?	Populations going to hospitals that were excluded from HD data Fewer hospital access in rural counties in Oregon may impact hospitalization decisions Populations living/working cross states border	3	Populations excluded from Oregon WC coverage (see Sensitivity section) Populations tended not to fine WC claims^22^, ^23^, ^24^	3	Population with nonoccupational lead exposure (tracked in the OHI) Population living/working cross state borders	4	3.3
Average score (representativeness)		…	4.0	…	4.0	…	4.5	4.2
Average overall score		…	3.7	…	4.3	…	4.3	4.1

Abbreviations: ABLES, adult blood lead epidemiology and surveillance; BLL, blood lead level; HD, hospital discharge; OHI, occupational health indicator; WC, workers’ compensation.

Overall, the Oregon OSH surveillance system data were fairly good in data quality and PVP (ratings: 4.3), but they had lower scores for sensitivity and representativeness (ratings: 3.6 and 4.2, respectively), due to under‐reporting and undercoverage among these data sources commonly reported in existing literature.^7^, ^17^, ^18^, ^19^, ^22^, ^23^ The ABLES data were rated relatively higher in sensitivity (rating: 4.0), considering the mandatory requirement of medical examination for lead‐exposed workers and the active case follow‐up in the ABLES system, which help to identify more true cases.^19^, ^25^

Among the three data sources, the disabling WC data and ABLES data had relatively higher overall score (ratings: 4.3). The inpatient HD data had the lowest score (rating: 3.7) due largely to the concerns of HD data quality issues reported in existing literature.^17^, ^18^

The overall average rating for the Oregon OSH surveillance system was 4.1, suggesting a relatively good overall data quality.

### 3.3 Overall usefulness

Stakeholders’ average rating of the relevance of the system's objectives and activities to the OSH needs was 4.1 on a 5‐point scale, with 93% rating it as 4 (relevant) or 5 (very relevant). Their average rating of the overall system's usefulness was 3.0, with 70% rating it as 3 (moderately useful) or below (somewhat useful/not useful). Despite the recognition that display of state‐level OHIs adds value to Oregon OSH profile, the Oregon OSH surveillance system, funded as an expanded program, had not demonstrated its usefulness to inform state and local‐level decision making (Table 6). A few main factors were identified as impacting the system's usefulness as discussed below.

**Table 6:** Evaluation results on the system's overall usefulness

Attributes	Evaluation measure	Evidence collected	Assessment	Overall evaluation
Usefulness	(1) Contribute to the prevention and control of adverse occupational health events and to an improved understanding of the public health implications of such events;	Lack of active data dissemination Lack of data usage OHIs are useful in tracking state‐level trends but limited in guiding local OSH practices The system has created little outcomes and impacts	No significant outcomes & impacts	Not useful
	(2) Relevance of system's activities to the OSH needs perceived by stakeholders	Average rating was 4.1.	Relevant	
	(3) Overall usefulness of the surveillance system perceived by stakeholders	Average rating was 3.0 Many stakeholders pointed out the importance of putting data into use	Moderate	

Abbreviations: OHI, occupational health indicator; OSH, occupational safety and health.

## 4 DISCUSSION

OSH surveillance collects data on work‐related health outcomes and hazards to identify populations at risk and guide intervention strategies to prevent workplace injury, illness and death. State‐level OSH surveillance programs are key to nationwide OSH surveillance in the US. OHI production is regarded as useful in helping states establish fundamental OSH surveillance capacity and contributing to a nationwide OSH profile. Once states establish fundamental surveillance capacity, they should take every opportunity to evaluate and enhance the quality of the surveillance system and to expand the usability of data it generates. Evaluation of OSH surveillance systems has rarely been reported in existing literature. This paper presents methods and findings in evaluating Oregon's OSH surveillance system and can be a reference for evaluations of other OSH surveillance systems. Limitations in applying the *CDC Updated Guidelines* on OSH surveillance evaluation were also discovered.

### 4.1 Factors limiting usefulness

The evaluation identified a few main factors limiting the Oregon OSH surveillance system's usefulness, including lack of timeliness of OHIs, lack of active data use and distribution, and the limited usability of OHIs in guiding local OSH practices.

*Time lag*: The OHIs are reported 2 to 3 years after the incidence of events or cases. In contrast, the reporting lag in other comparable public health surveillance (eg, chronic diseases) was usually around 18 months (6 months after the end of each calendar year).^27^ Many health outcome data sources used for calculating OHIs were fairly timely, such as the disabling WC claims data, the HD data, and ABLES data. However, some of the denominator data, such as the US Census data, have a much longer lag time and thus affect the timeliness of OHIs.

The importance of timeliness varies depending on the surveillance purposes and the practicality to guide actions. Most stakeholders (70%) accepted the time lag given OHIs are lagging indicators by nature. The CSTE work group developed OHIs to help states build OSH surveillance capacity and contribute to national OSH surveillance efforts. To facilitate comparison between states, easy access to state‐wide data for most states was a critical consideration in OHI design.^7^ However, the long lag limits OHIs’ ability to reflect emerging OSH issues, to guide timely interventions and practices, and to measure current progress and effectiveness of OSH programs. Stakeholders pointed that some OHIs could be more useful if they were more timely. For example, timely reporting of OHI on influenza vaccination coverage among health care personnel could guide preparation for flu seasons. Some OHIs could be more timely as new data sources were becoming available, such as the data of emergency department (ED) visits. In fact, states could calculate and act on individual OHIs with timelier data beyond the production of the entire annual OHI report.

*State vs substate scale*: While calculation of state‐level OHIs helps describe OSH variations between states, it limits the usability of OHIs for state OSH programs to focus efforts within states. OHIs as currently calculated lack substate level information on factors such as demographics, industry and occupation, and geographical locations. As such, they cannot identify local risks and populations at risk. States could work with partners to develop disaggregated OHIs with local‐level information.

*Data dissemination and data use*: As pointed out in other surveillance evaluations, broader data dissemination is an important way to improve surveillance usefulness.^28^, ^29^ Although OOPHP produces an annual OHI publication, there has been disincentive to promote OHI data. Stakeholders commented that they did not think that “this data is widely published or leveraged.” Program leadership and key staff identified a few issues impacting data dissemination and use. First, they were unsure about how OHIs could be used to guide prevention practices due to the long lag and lack of substate level data. As such, OOPHP had difficulty in targeting end users who may use the information and recommendations for prevention interventions. More generally, OOPHP lacks appropriate staff resources, such as a health educator, to develop and distribute tailored outreach materials to promote occupational public health interventions. Similar to many other surveillance systems, limited resources (eg, funding and staffing time) challenge the program's capacity to conduct more ambitious activities, including active data dissemination.^29^, ^30^

The usefulness of public health surveillance relies on the effective production and use of data to improve health research and practice. Given the OHIs’ scale and timeliness limitations and resulting lack of effective data, the Oregon OSH surveillance system did not demonstrate its usability among end users.

### 4.2 Recommendations and improvement actions

Based on the above findings, to improve the OSH surveillance system, OOPHP should explore existing and new data sources that complement those specified in the CSTE OHI guide with more local context to produce demographic, employment and hazard‐specific data and timelier indicators that are more responsive to OSH needs in the state. The future of public health surveillance and OSH surveillance includes the use of multiple emerging data sources, including rapidly evolving health care and nonhealth information systems.^2^, ^31^ Emerging data sources such as Oregon Oregon ESSENCE (the Electronic Surveillance System for the Early Notification of Community‐Based Epidemics, a syndromic surveillance system which captures ED visit data and urgent care data within hours) and other electronic health records data promises timelier and more comprehensive tracking of work‐related injury and illness.^32^, ^33^, ^34^, ^35^

A very few of the 24 OHIs lend themselves to timely substate level data generation. To promote surveillance data usage, OOPHP needs to develop interpretable information to suit users’ needs and effectively disseminate this information via outreach and engagement of end users. A good example is the county‐level OHIs in story map form that portrays the areas of Oregon with more workers in high‐risk industry sectors and associated higher levels of occupational injury and illness using an interactive online platform.^16^ The project team consulted the technical guidance on substate measures released by the CSTE, which aims to guide states on optional measures at county and regional level based on existing OHIs.^36^ The system could further this type of work by continuing to use such resources and guides based on Oregon's OSH conditions and needs.

Fostering wide collaboration with different public health agencies, research institutions, and organizations within and outside the State of Oregon could help to obtain more resources for surveillance and data dissemination. Integrating OSH surveillance into broader public health initiatives and goals relating to chronic disease, communicable disease, injury and violence prevention, and other disciplines could be one way to advance occupational public health interventions.

Based on identified gaps and evaluation recommendations, OOPHP is making improvements. For example, the program is exploring the use of syndromic surveillance data and ED visits data to supplement existing OHIs. OOPHP is also planning on further improvement actions.

### 4.3 Lessons from the *CDC updated guidelines*

During this project, the evaluators learned that the *CDC Updated Guidelines* did not sufficiently guide OSH surveillance evaluation. The *CDC Updated Guidelines* has been criticized as being designed towards communicable disease surveillance and not always applicable to different types of surveillance.^9^, ^10^ Some attributes and example measures have less relevance for OSH surveillance. For example, stability is defined in the guidelines as “no failure in operating the system” and “the system being operational when needed.” This is important to systems in which operation failures could impede public health actions that require quick action, such as infectious disease outbreak detection and response. Such measures are less relevant to many current OSH surveillance systems which focus on using existing data sources to inform of careful interventions, rather than quick action.

Due to lack of guidance on weighting attributes in the *CDC Updated Guidelines* and many other common guidelines, we chose to treat attributes and measures equally in terms of their impacts on the overall system performance. In fact, studies have shown that some attributes and components may play a more important role in a surveillance system.^37^, ^38^ For example, organizational drivers such as resource availability, training, organization and management remarkably impact performance related attributes. Meanwhile, attributes like acceptability, data completeness and correctness are central in relation to many other attributes.^38^ Identifying and assigning larger weights to attributes that have more impact and/or are more central in the OSH surveillance system can help to more precisely pinpoint the system's performance and target important areas.

Many existing guiding approaches including the *CDC Updated Guidelines* provide only general recommendations, which are not enough to guide a comprehensive evaluation.^8^ Further, to the best of our knowledge, there has been no published guidelines tailored for OSH surveillance evaluation. Although the evaluation team was able to develop tactics and evaluation methods for this project, the lack of detailed guidance created challenges. A framework tailored to OSH surveillance with more specific guidance is needed to facilitate evaluation of this type of public health surveillance. The tailored framework could include attributes and measures suitable for OSH surveillance systems, as well as weights of attributes and measures to indicate their importance.

### 4.4 Study limitations

Limited by available time and resources for the evaluation, the evaluation team conducted primarily qualitative assessment of data quality attributes and limited the evaluation to selected data sources. Quantitative analysis on data quality attributes such as sensitivity and specificity was not performed. The evaluators felt that it is infeasible to include quantitative data quality assessment in a routine surveillance evaluation given the time and toolkits needed. Special studies are required for more thorough analysis on data quality.

The evaluation team identified a comprehensive list of stakeholders and actively sought their participation. Selection bias might exist on the part of the participating stakeholders since they may hold a more positive attitude towards the system. We noticed that the online survey had relatively low response rate and stakeholders who did not respond tended to less actively participate in the program's routine activities. This indicates a challenge in the evaluation to engage stakeholders with lower level of involvement. Few data users were identified or included in this evaluation due to the lack of data usage. However, a strength of the evaluation was the use of multiple information sources to collect evaluation evidence. Therefore, bias from stakeholders could be effectively minimized.

## 5 CONCLUSION

OOPHP has reported OHIs since 2004 to track trends in major occupational injuries, illnesses, deaths, and hazards at a state‐wide level. A comprehensive evaluation conducted in 2018 found that overall the OSH surveillance system has many positive attributes. The system was very simple and highly accepted by its stakeholders. It was flexible in accommodating changes related to OHI and other surveillance activities. The system is stable, however a lack of resources and long‐term funding security present challenges to improving surveillance and program sustainability. Assessment of three key data sources showed the surveillance data had fairly good quality but was relatively poor regarding sensitivity and representativeness. The lack of timeliness and usability of OHIs in guiding local OSH practices creates a disincentive for active data dissemination, resulting in a lack of usefulness of the Oregon OSH surveillance system. OOPHP should enhance the capacity of its surveillance system to use existing and new data sources to produce timely, substate level information that describe local occupational health burdens and disparities, promote active data dissemination, and foster collaborations to promote occupational public health interventions.

This evaluation identified limitations of the *CDC Updated Guidelines* for evaluating OSH surveillance systems. There was no detailed guidance on how to select relevant attributes and measures and assign weights to them. A future tailored framework with more specific guidance will guide better evaluation of OSH surveillance systems. Further research is needed to develop such a guiding framework and to promote more evaluations on OSH surveillance.

## CONFLICT OF INTERESTS

Liu Yang was funded by the Oregon's Occupational Public Health program to attend the Council of State and Territorial Epidemiologists annual conference (Raleigh, NC, 2‐6 June 2019). Each author has completed the conflict of interest form.

## DISCLOSURE BY AJIM EDITOR OF RECORD

John D. Meyer declares that he has no conflict of interest in the review and publication decision regarding this article.

## AUTHOR CONTRIBUTIONS

LY substantially worked on conception and design of this project, as well as the acquisition, analysis, and interpretation of data. CW, CC, and LK substantially contributed to the design of the study, acquisition, analysis, and interpretation of data. LY drafted the manuscript. All authors substantially worked on manuscript revisions. All authors gave final approval to be published and agree to be accountable for all aspects of the work.

## ETHICS APPROVAL AND INFORMED CONSENT

The work was performed with the Public Health Division at Oregon Health Authority. No ethics review and approval were required because the project was regarded as evaluation instead of research.

## Supporting information

Supporting information
