# Supplementary material for: Evaluating Oregon's occupational public health surveillance system based on the CDC updated guidelines
Source: Am J Ind Med. 2020 Jun 1;63(8):713–25. doi: 10.1002/ajim.23139 (PMC7383881; doi:10.1002/ajim.23139)
Supplement: Supplementary file 1 — Supporting information [file AJIM-63-713-s001.docx]

# Appendix: Evaluation Guides and Questions

**Appendix 1. Interview guide for higher level leaders**

1. What is the importance and significance of the OOPHP/OSH surveillance system?
2. How does the OOPHP/OSH surveillance system fit into the organization’s objectives and goals?
3. What is the long-term strategic plan in your organization to further support the system?
4. What are the challenges or barriers for the organization to support the system?
5. What are your suggestions to further improve the OSH surveillance system?

**Appendix 2. Interview guide for key OSH surveillance staff**

1. Please describe the working process/steps including data request, and the approximate time spent for each step in the OSH surveillance process?
2. For each OHI, please rate how simple it is to identify cases on a 5-point scale, with 1 indicating not simple and 5 the simplest.
3. For OHIs you rated 1 or 2, please explain the reasons.
4. How many organizations request work reports and the surveillance data on a regular basis?
5. Have there been any problems on adapting changes in OHI surveillance guide or other changes?
6. Are there formal trainings on knowledges and skills needed for the OSH surveillance?
7. How quickly and timely the data providers and other collaborators respond to your work requests?
8. How do you think of the overall data quality in the system?
9. Do you have any concerns on the completeness, validity, sensitivity, or PVP of the data in this system?
10. Are you aware of any study done on validity, sensitivity, or PVP for data in this system?
11. Do you think that certain sub-populations in Oregon are excluded/under-reported in the OHIs?
12. Have you experience any failure in the work infrastructure that caused you not being able to complete the surveillance work in time?
13. Are there any potential changes/challenges with data availability and the surveillance infrastructure that could be a threat to the stability of the OSH surveillance system?

**Appendix 3. Interview guide for data providers**

1. Please briefly describe how you process your data from obtaining the raw data to releasing the data.
2. Please describe the data quality control process in place to monitor errors and missing values in your system.
3. Do you have protocols on the data quality control process?
4. Do you have formal training on data processing and data quality control?
5. Please describe the process dealing with the OSH surveillance system’s data request, and the approximate time it takes.
6. How do you think of the overall data quality in your system?
7. Do you have any concerns on the completeness, validity, sensitivity, or PVP of the data in your system?
8. Do you have completeness rate data of the key variables the OSH surveillance system needed? If yes, could you show me?
9. Are you aware of any study done on validity, sensitivity, or PVP for data in your system?
10. Do you think that certain sub-populations in Oregon are excluded/under-reported in your data?
11. Are there any active surveillance approaches in place to increase the sensitivity and/or correct errors?
12. Do you expect major technical changes in the next 5 years for your data system? If yes, please briefly introduce them.
13. Do you expect major other changes in the next 5 years in your system, which may impact the collaboration between you and the OSH surveillance system? If yes, please briefly introduce them.
14. Do you have any suggestions for us to better collaborated with your agency?

**Appendix 4. Focus group discussion guide**

1. Can you think of any potential future challenges/opportunities that may impact our occupational health surveillance system?
2. What strategies/methods should the program use to respond to these challenges?
3. What is your opinion regarding the 2 to 3 years’ time lag in OHIs? Is it OK for guiding occupational safety and health (OSH) practices?
4. Are there any specific occupational hazards would benefit from being captured quickly?
5. Are there any barriers for a shorter time lag?
6. What are possible ways that we could use OHI surveillance data?
7. What are the intended end users of the data? And how we could reach out to them?
8. What are possible disseminating channels that we could use?

**Appendix 5. Survey questions**

1. On a scale of 1 to 5, please rate your agency's or your willingness in collaborating with the OOPHP.
2. Please let us know if there is any challenges or barriers for your agency or you to collaborate with the OOPHP.
3. Are you aware of the objective(s) of the OSH surveillance system?
4. Do you think the program's objective and activities are relevant to the needs of occupational safety and health in Oregon? (5-Likert scale, objectives provided)
5. Please let us know the reason(s) why you think it's relevant/not relevant. Any suggestions to make it more relevant?
6. Overall, how useful do you think the Oregon OPHP occupational health surveillance system is, in terms of guiding occupational safety and health practices? (5-Likert scale)
7. Do you have any additional suggestions for us to improve the Oregon OSH surveillance system?

**The following questions are only for management and key personnel and external experts:**

1. What is your opinion regarding the 2 to 3 year’s time lag in OHIs? Do you think it is OK for guiding occupational safety and health practices? And why?
2. Are there any barriers for a shorter time lag in OHIs? (only for management and key personnel)
3. Which indicator(s) might benefit if we were able to acquire more timely data? And why?
4. In OSH field, what challenges or opportunities do you think exist or will emerge in the future, which may have significant impact on the Oregon OSH surveillance? How should the system respond to them? (only for OOPHP advisory committee members)
